# The Impact of Influenza Vaccinations on the Adverse Effects and Hospitalization Rate in the Elderly: A National Based Study in an Asian Country

**DOI:** 10.1371/journal.pone.0050337

**Published:** 2012-11-28

**Authors:** Tsung-Yu Ho, Kuang-Yung Huang, Tza-Ta Huang, Yung-Sung Huang, Hsu-Chueh Ho, Pesus Chou, Chun-Hung Lin, Chang-Kao Wei, Wei-Chang Lian, Ting-Chang Chen, Hsien-Bin Huang, Ching-Chih Lee

**Affiliations:** 1 Department of Otolaryngology, Buddhist Dalin Tzu Chi General Hospital, Chiayi, Taiwan; 2 Divsion of Neurology, Department of Internal Medicine, Buddhist Dalin Tzu Chi General Hospital, Chiayi, Taiwan; 3 Division of Allergy, Immunology, and Rheumatology, Department of Internal Medicine, Buddhist Dalin Tzu Chi General Hospital, Chiayi, Taiwan; 4 School of Medicine, Tzu Chi University, Hualian, Taiwan; 5 Community Medicine Research Center and Institute of Public Health, National Yang-Ming University, Taipei, Taiwan; 6 Department of Surgery, Buddhist Dalin Tzu Chi General Hospital, Chiayi, Taiwan; 7 Division of Metabolism and Endocrinology, Department of Internal Medicine, Buddhist Dalin Tzu Chi General Hospital, Chiayi, Taiwan; 8 Cancer Center, Buddhist Dalin Tzu Chi General Hospital, Chiayi, Taiwan; 9 Department of Life Science and Institute of Molecular Biology, National Chung Cheung University, Chiayi, Taiwan; 10 Department of Oral and Maxillofacial Surgery, Changhua Christian Hospital, You-Lin Branch, You-Lin, Taiwan; Fudan University, China

## Abstract

**Objectives:**

To examine the risk of adverse effects of special interest in persons vaccinated against seasonal influenza compared with unvaccinated persons aged 65 and above.

**Methods:**

We retrospectively observed 41,986 vaccinated elderly persons and 50,973 unvaccinated elderly persons in Taiwan from October 1, 2008, through September 30, 2009, using the National Health Insurance database. Neurological and autoimmune disorders and one-year hospitalization rates and in-hospital mortality rates were analyzed according to the vaccination status. Propensity score analysis was used to assess the relationship between adverse outcomes, hospitalization rates, and vaccination status.

**Results:**

45% of the elderly received influenza vaccination. Multiple logistic regression showed that the probability of being vaccinated was related to more patients visiting for URI symptoms (odds ratio (OR), 1.03; 95% CI, 1.02–1.03), men (OR, 1.15; 95% CI, 1.12–1.17), increased age (OR, 1.02; 95% CI, 1.02–1.03), and more comorbidities (OR, 1.2; 95% CI, 1.17–1.23). There were no statistical differences in neurological and autoimmune diseases between the vaccinated and unvaccinated individuals using propensity score analysis, but vaccinated persons had a reduced hospitalization rate of 19% (odds ratio [OR], 0.81; 95% CI, 0.77–0.84) for the first six-months and 13% for one-year of follow-up (OR, 0.87; 95% CI, 0.85–0.9).

**Conclusions:**

Based on data from the one-year follow-ups among 93,049 elderly persons in Taiwan, reassuring results for selected neurological and autoimmune diseases were found among the vaccinated individuals after adjusting other factors. Influenza vaccination decreased the risk for hospitalization. Public health strategies must continue to improve the influenza vaccination rate among the elderly with information based upon tangible evidence.

## Introduction

Influenza, which typically occurs during the late fall through early spring in the United States, is associated with high rates of morbidity and mortality, especially for the elderly [1]. Approximately 36,000 deaths from 1990 to 1999 and 226,000 hospitalizations from 1979 to 2001 were associated with influenza epidemics [2], [3]. Annual influenza vaccinations have been proven to prevent influenza infection, prevent further complications from influenza, reduce hospitalization rates, and shorten hospital stays among the elderly [4], [5].

Although the protective effect of influenza vaccinations have been proven, concern about influenza vaccination safety is often cited by the mass media or persons who decline to undergo vaccination. Several neurological disorders have been reported to be associated with influenza vaccinations. Bell's palsy, Guillain-Barre syndrome (GBS), pericarditis, and nephritic syndrome have been noted in individuals with influenza vaccination in case reports [6], [7], [8]. This is especially important for GBS, a relatively rare condition that was causally linked to the 1976 swine-influenza A (H1N1) vaccination campaign in the United States [9]. Results from the development of H5N1 vaccines revealed that adjuvants may reduce the amount of antigen needed to create an adequate immunological response which may further provoke autoimmune diseases [10], [11], [12]. A recent study revealed mild increased risk for Bell's palsy, paraesthesia, and inflammatory bowel disease was noted in individuals with H1N1 influenza vaccinations in Sweden [13]. However, detailed data for autoimmune diseases and neurological disorders in Asian countries remain unknown. In order to achieve higher influenza vaccination coverage for persons aged 65 years or above, adverse events risk evaluation and new strategies may be needed [14].

Given the suggestive but limited data on influenza vaccinations and the risk of autoimmune diseases and neurological disorders, we conducted a retrospective cohort study using the National Health Insurance (NHI) Research Database in Taiwan, a unique nationwide, population-based claims data system with information on the medical history of all citizens.

**Table 1:** Numbers and percentage proportions of vaccine coverage by sex, comorbidities, geographic regions and urbanization of residence, and individual socioeconomic status (n = 93049).

	Vaccinated (n = 41986) n (%)	Unvaccinated (n = 51063) n(%)	Coverage (%)	P value
All	41986	51063	45.1	
Age, year	75±6.5	74±6.9		
Age group (years)				<0.001
65–70	12912	21075	38.0	
71–75	11032	11807	48.3	
76–80	9864	9102	52.0	
>80	8178	9079	47.4	
Gender				<0.001
Female	21185	27587	43.4	
Male	20801	23476	47.0	
URI OPD# in prior 6 months (mean±SD)	1.55±2.98	1.32±2.77		<0.001
Number of Comorbidities				<0.001
0	19392	26203	42.5	
1	15395	17566	46.7	
2	5965	6131	49.3	
> = 3	1234	1163	51.5	
Geographic region				<0.001
Northern	16118	23019	41.2	
Central	12452	12775	49.4	
Southern	12277	13732	47.2	
Eastern	1139)	1537	42.6	
Urbanization				<0.001
Urban	9004	13030	40.9	
Suburban	15617	19515	44.5	
Rural	17365	18518	48.4	
Socioeconomic status				<0.001
High	429	836	33.9	
Medium	16613	17696	48.4	
Low	24944	32531	43.4	

## Materials and Methods

### Ethics statement

This study was initiated after approval by the Institutional Review Board of Buddhist Dalin Tzu Chi General Hospital, Taiwan. Since all identifying personal information was stripped from the secondary files before analysis, the review board waived the requirement for written informed consent from the patients involved.

**Table 2:** Predictors for influenza vaccination in the elderly (n = 93049).

	Adjusted odds ratio	(95% CI)	*P* value
URI OPD# in prior 6 months	1.03	(1.02–1.03)	<0.001
Gender			
Reference: Female			
Male	1.15	(1.12–1.17)	<0.001
Age (years)	1.02	(1.02–1.03)	<0.001
Comorbidity			
Reference: No			
Yes	1.20	(1.17–1.23)	<0.001
Geographic region			
Reference: Northern			
Central	1.31	(1.26–1.36)	<0.001
Southern	1.15	(1.11–1.20)	<0.001
Eastern	0.94	(0.87–1.02)	0.133
Urbanization			
Reference: Rural			
Suburban	0.90	(0.87–0.93)	<0.001
Urban	0.87	(0.84–0.91)	<0.001
Socioeconomic status			
Reference: Low			
Medium	1.10	(1.07–1.14)	<0.001
High	0.72	(0.64–0.81)	<0.001

95% CI, 95% confidence interval.

### Database

The National Health Insurance program, which provides compulsory universal health insurance, was implemented in Taiwan in 1995. It enrolls up to 99% of the Taiwanese population and contracts with 97% of all medical providers. The database contains comprehensive information on insured subjects, including dates of clinical visits, diagnostic codes, details of prescriptions, and expenditure amounts. This study used the Longitudinal Health Insurance Dataset for 2008–2009 released by the Taiwan National Health Research Institute. The patients studied did not significantly differ statistically from the larger cohort in age, gender, or health care costs, as reported by the Taiwan National Health Research Institute (www.nhri.org.tw) [15], [16].

**Table 3:** Adverse events and hospitalization rates among the study population for one-year of follow-up*.

Diseases and vaccination status	Follow-up period												
	1^st^ month	2^nd^ month	3^rd^ month	4^th^ month	5^th^ month	6^th^ month	7^th^ month	8^th^ month	9^th^ month	10^th^ month	11^th^ month	12^th^ month	
	(2008/10)	(2008/11)	(2008/12)	(2009/1)	(2009/2)	(2009/3)	(2009/4)	(2009/5)	(2009/6)	(2009/7)	(2009/8)	(2009/9)	Total
Bell's palsy													
Unvaccinated	4(0.01)	7(0.01)	7(0.01)	8(0.02)	3(0.01)	5(0.01)	6(0.01)	6(0.01)	3(0.01)	5(0.01)	2(0.004)	5(0.01)	61(0.12)
Vaccinated	6(0.01)	9(0.02)	8(0.02)	4(0.01)	5(0.01)	3(0.01)	7(0.02)	8(0.02)	4(0.01)	7(0.02)	4(0.01)	3(0.01)	68(0.16)
Multiple sclerosis													
Unvaccinated	1(0.002)	0	1(0.002)	0	0	1(0.002)	1(0.002)	1(0.002)	0	1(0.002)	0	0	6(0.012)
Vaccinated	0	0	0	1(0.002)	0	0	0	1(0.002)	0	0	0	0	2(0.005)
An/hypoesthesia, Paraesthesia													
Unvaccinated	2(0.004)	1(0.002)	1(0.002)	1(0.002)	2(0.004)	1(0.002)	4(0.008)	2(0.004)	1(0.002)	5(0.01)	4(0.008)	3(0.006)	27(0.053)
Vaccinated	4(0.01)	1(0.002)	2(0.005)	2(0.005)	3(0.007)	1(0.002)	1(0.002)	1(0.002)	0	2(0.005)	1(0.002)	6(0.014)	24(0.057)
Guillain-Barré syndrome													
Unvaccinated	1(0.002)	0	1(0.002)	0	2(0.004)	1(0.002)	1(0.002)	3(0.006)	0	1(0.002)	1(0.002)	1(0.002)	12(0.024)
Vaccinated	0	0	0	0	2(0.005)	3(0.007)	2(0.005)	2(0.005)	1(0.002)	2(0.005)	3(0.007)	2(0.005)	17(0.040)
Sudden hearing loss													
Unvaccinated	2(0.004)	0	1(0.002)	1(0.002)	0	1(0.002)	1(0.002)	1(0.002)	1(0.002)	1(0.002)	0	1(0.002)	10(0.02)
Vaccinated	1(0.002)	1(0.002)	0	0	0	2(0.005)	0	2(0.005)	0	0	1(0.002)	0	7(0.02)
Rheumatoid arthritis													
Unvaccinated	1(0.002)	1(0.002)	0	0	0	3(0.006)	2(0.004)	1(0.002)	3(0.006)	1(0.002)	1(0.002)	3(0.006)	16(0.03)
Vaccinated	0	2(0.005)	3(0.007)	0	1(0.002)	3(0.007)	0	2(0.005)	1(0.002)	1(0.002)	1(0.002)	3(0.007)	17(0.04)
Type I diabetes													
Unvaccinated	5(0.01)	5(0.01)	5(0.01)	3(0.006)	9(0.018)	5(0.01)	5(0.01)	12(0.024)	10(0.02)	12(0.024)	7(0.014)	5(0.01)	83(0.163)
Vaccinated	2(0.005)	2(0.005)	6(0.014)	2(0.005)	6(0.014)	4(0.01)	3(0.007)	8(0.019)	6(0.014)	6(0.014)	6(0.014)	7(0.017)	58(0.138)
Hospitalization for all causes													
Unvaccinated	701(1.37)	733(1.44)	1033(2.02)	1174(2.29)	1104(2.16)	1004(1.97)	862(1.69)	847(1.66)	801(1.57)	815(1.59)	719(1.41)	658(1.29)	40451(20.47)
Vaccinated	320(0.76)	551(1.31)	808(1.92)	903(2.15)	867(2.06)	822(1.96)	760(1.81)	678(1.61)	669(1.59)	704(1.68)	667(1.59)	569(1.36)	8318(19.81)

* The percentage was expressed in parentheses.

### Study Population

All patients with a recorded age of 65 and above in the dataset between October 1, 2008 and Dec 31, 2008 were included. We excluded those with any type of adverse outcomes, such as Bell's palsy, multiple sclerosis, Guillain-Barre syndrome, hypoesthesia, sudden hearing loss, rheumatoid arthritis, and type I diabetes.

**Figure 1:**
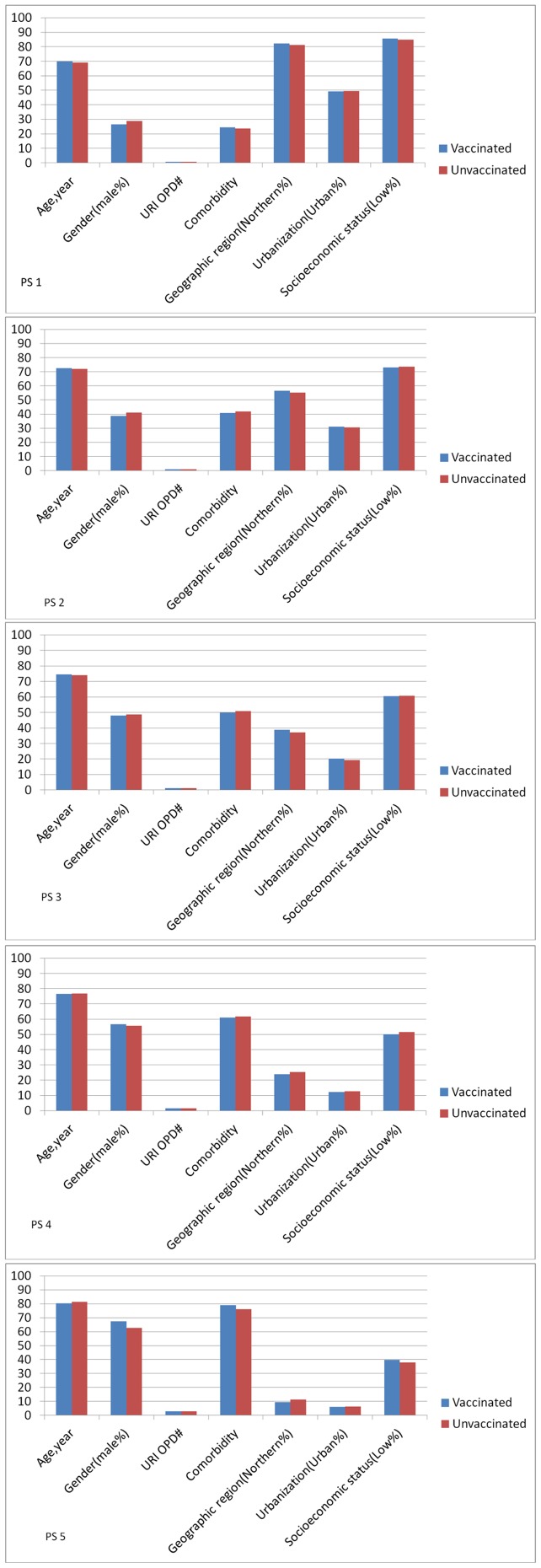
Distribution of explanatory variables between patients receiving influenza vaccine and those not receiving influenza vaccine for propensity score quintiles ranging from 1 (least likely to receive influenza vaccine) to 5 (most likely to receive influenza vaccine).

**Table 4:** Risk of selected neurological and autoimmune diseases and hospitalization in vaccinated versus unvaccinated groups (n = 93049).

Disease	Vaccinated (n = 41986) n (%)	Unvaccinated (n = 51063) n (%)	Mantel-Haenszel adjusted odds ratio (95% CI)	*P* value*
***For the first six-months of follow-up***				
**Neurological diseases**				
Bell's palsy	37 (0.09)	35 (0.07)	1.30 (0.82–2.07)	0.248
Multiple sclerosis	1 (0.002)	3 (0.01)	0.38 (0.04–3.59)	0.374
Guillain-Barré syndrome	5 (0.01)	5 (0.01)	1.19 (0.34–4.20)	0.794
An/hypoesthesia, Paraesthesia	13 (0.03)	8 (0.02)	2.07 (0.84–5.12)	0.115
Sudden hearing loss	4 (0.01)	5 (0.01)	0.95 (0.25–3.65)	0.945
**Autoimmune diseases**				
Rheumatoid arthritis	9 (0.02)	5 (0.01)	2.42 (0.80–7.30)	0.106
Type I diabetes	22 (0.05)	32 (0.06)	0.81 (0.47–1.40)	0.441
**Hospitalization**				
Hospitalization for all causes	4271 (10.17)	5749 (11.26)	0.81 (0.77–0.84)	<0.001
***For one-year of follow-up***				
**Neurological diseases**				
Bell's palsy	68 (0.16)	61 (0.12)	1.39 (0.98–1.97)	0.058
Multiple sclerosis	2 (0.004)	6 (0.01)	0.35 (0.07–1.77)	0.188
Guillain-Barré syndrome	17 (0.04)	12 (0.02)	1.64 (0.77–3.49)	0.198
An/hypoesthesia, Paraesthesia	24 (0.06)	27 (0.05)	2.07 (0.84–5.12)	0.115
Sudden hearing loss	7 (0.02)	10 (0.02)	0.95 (0.25–3.65)	0.945
**Autoimmune diseases**				
Rheumatoid arthritis	17 (0.04)	16 (0.03)	1.39 (0.69–2.79)	0.356
Type I diabetes	58 (0.14)	83 (0.16)	0.84 (0.60–1.17)	0.301
**Hospitalization**				
Hospitalization for all causes	8318 (19.81)	10451 (20.47)	0.87 (0.85–0.90)	<0.001

* P value for Mantel-Haenszel chi-square test.

95% CI, 95% confidence interval.

A total of 93,049 individuals were included. Each patient was tracked from his or her index ambulatory visit between October 1, 2008 and September 30, 2009 to identify outcomes. These patients were then linked to the administrative data for the 2008 to 2009 period to calculate cumulative risk, with cases censored for patients who drew back guarantees from the National Health Insurance Program or were still healthy without defined events at the end of follow-up.

### Exposure to influenza vaccinations

In this series, vaccinations were paid for by the Department of Health and given without charge to the elderly in Taiwan. The vaccines were Fluvirin (Navartis Vaccine) , Vaxigrip (Pasteur Merieux Connaught) and Fluarix (Glaxo SmithKline). Fluvirin, Vaxigrip and Fluarix are inactivated, split, not-adjuvanted, trivalent, and seasonal influenza vaccines. A 0.5 mL dose contains 15 mcg each of antigens similar to those of A/Brisbane/59/2007 (H1N1), A/Brisbane/10/2007 (H3N2), and B/Florida/4/2006. The type and amount of viral antigens contained in Fluvirin, Vaxigrip or Fluarix conform to the United States Public Health Services (USPHS) requirements and World Health Organization (WHO) recommendations for the season. The free vaccination program ran from October 1 through December 31, 2008.

### Definitions of neurological and autoimmune diagnoses

The neurological and autoimmune diagnoses for follow-up were in line with the previous study and the European Medicines Agency strategy for monitoring the safety of the pandemic vaccines, which was defined according to the ICD-9 codes for hospitalization and ambulatory visits [13], [17]. Outcomes from the hospitalization and ambulatory visits records include Bell's palsy (ICD-9-CM 351.0–351.1), Guillain-Barre syndrome (ICD-9-CM 357.0, 357.81, 357.89, 357.9), and sudden hearing loss (ICD-9-CM 388.2). The diagnosis of Guillian-Barre syndrome was verified by neurology specialists. An/hypoesthesia or paraesthesia (ICD-9-CM 780.2) were extracted from the ambulatory visits records. Diagnoses of multiple sclerosis (ICD-9-CM 340, 341.0, 341.9), rheumatoid arthritis (ICD-9-CM 714.0 ) and type I diabetes (ICD-9-CM 250.01, 250.11–250.13, 250.23, 250.31, 250.33, 250.41, 250.43, 250.51, 250.53, 250.61, 250.63, 250.71, 250.73, 250.81, 250.83, 250.91, 250.93) were confirmed by the catastrophic disease dataset released from the NHIRD.

### Definitions of hospitalization

Hospitalization for all causes between the two cohorts during the study period were recorded [4].

### Definitions of covariates

Patients were characterized by age, gender, number of outpatient visits for URI during the six months before the vaccination date (for the vaccinated group) or the first day when the free vaccination was offered (for the unvaccinated group), comorbidities, geographic region and urbanization of residence, and individual socioeconomic status (SES). The comorbidities of each patient included existing heart disease, lung disease, diabetes, stroke, renal disease, and catastrophic illnesses (cancer and rare diseases) [18]. Baseline comorbid conditions were defined as having any inpatient or outpatient diagnosis of the above mentioned diseases during the preceding year prior to October 1, 2008.

This study used income-related insurance payment amount as a proxy measure of individual SES at the time of diagnosis. The individuals were classified into three groups: (1) low SES: lower than US$571 per month (New Taiwan Dollar (NT$) 20,000); (2) moderate SES: between US$571-1,141 per month (NT$20,000-40,000); and (3) high SES: US$1,142 per month (NT$40,001) or more [19]. The geographic regions and urbanization of residence were classified as previously described [20], [21].

### Statistical analysis

The SAS statistical package, version 9.2 (SAS Institute, Inc., Cary, NC), and SPSS version 15 (SPSS Inc., Chicago, IL) were used for data analysis. Pearson's chi-square test was used for categorical variables, demographic characteristics (gender, region, and urbanization of residence), comorbidities, and individual socioeconomic status in vaccinated and unvaccinated individuals. Continuous variables, such as age, and URI OPD# were analyzed with two-sample t-tests in this series. Multiple logistic regression was used to estimate the predictors for receiving a flu vaccination based on each individual's observed variables, which included URI OPD# in the prior six months, gender, age, comorbidity, geographic region and urbanization of residence, and individual SES.

The cumulative risk of neurological outcomes/autoimmune diseases was estimated as a function of time from initial treatment. To adjust for the potential bias caused by self-selection, propensity score was used [22]. To obtain the propensity score in this study, patient characteristics were entered into a logistic regression model to predict selection for vaccination. These characteristics were age, gender, URI OPD# in the prior six months, comorbidity, geographic region and urbanization level of residence, and socioeconomic status. The effect of influenza vaccination on the selected neurological and autoimmune diseases was analyzed with each quintile. The Mantel-Haenszel odds ratio was estimated in addition to performing the Cochran-Mantel-Haenszel χ^2^ test.

In order to clarify the benefit of vaccination, the odds ratio of hospitalization for all cases of vaccinated and unvaccinated individuals was performed using propensity score analysis. A *p<*0.05 was considered statistically significant in the regression models.

## Results

By December 31, 2008, virtually all vaccination activity had been completed, with a cumulative 41,986 people vaccinated (45.1% of the study population). Table 1 shows the vaccine coverage rate and distribution of demographic characteristics, URI OPD#, comorbidities, geographic regions and urbanization of residence, and socioeconomic status for the two cohorts. Individuals aged 71 and above were more often vaccinated than those aged 65 to 70 years. More men than women were vaccinated (46.9% vs. 43.4%). Individuals with more comorbidities were more likely to be vaccinated. Vaccine coverage was greater in those who lived in central and southern Taiwan (47.2–49.7% vs. 41.2–42.6%), in rural areas (48.4% vs. 43%), and those of medium and low socioeconomic status (43.4–48.4% vs. 33.9%).

Table 2 shows the predictors for receiving an influenza vaccine. Visitors of an outpatient clinic for URI before the study period in the prior six months were more likely to receive an influenza vaccine (odds ratio (OR), 1.03; 95% CI, 1.02–1.03). Male gender (OR, 1.15; 95% CI, 1.12–1317) and old age (OR, 1.02; 95% CI, 1.02–1.03) were more likely to be vaccinated. Individuals with more comrobidies were more likely to be vaccinated than those without any comorbidities (OR, 1.2; 95%, 1.17–1.23). Compared with those in northern Taiwan, individuals who lived in central (OR, 1.31; 95% CI, 1.26–1.36) and southern Taiwan (OR, 1.15; 95% CI, 1.11–1.12) were more likely to receive a flu vaccination. Residence in suburban (OR, 09; 95% CI, 0.87–0.93) and urban areas (OR, 0.87; 95% CI, 0.84–0.91) were negative predictors for receiving vaccination. Furthermore, individuals with moderate SES were more likely to be vaccinated (OR, 1.1; 95% CI, 1.07–1.14), compared with those with low SES.

Table 3 shows the adverse outcomes between the vaccinated and unvaccinated individuals. Among the neurological and autoimmune disease, seasonal preponderance was found in GBS among the individuals with or without vaccination: 41% of GBS patients in the vaccinated group and 42% of GBS patients in the unvaccinated group developed the disease in spring (March to May).

### Univariate analysis

The risks of neurological and autoimmune diseases were further analyzed according the first six-months and one-year of follow-up after the vaccination campaign. During the first six-months of follow up, there was no statistical difference in GBS (p = 0.144), Bell's palsy (p = 0.083), multiple sclerosis (p = 0.218), an/hypoesthesia, or paraesthesia (p = 0.848), sudden hearing loss (p = 0.744), rheumatoid arthritis (p = 0.461), and type I diabetes (p = 0.342) between the two groups. There was also no statistical difference in GBS (p = 0.144), Bell's palsy (p = 0.083), multiple sclerosis (p = 0.218), an/hypoesthesia, or paraesthesia (p = 0.848), sudden hearing loss (p = 0.744), rheumatoid arthritis (p = 0.461), and type I diabetes (p = 0.342) between the two groups after one-year of follow up in univariate analysis. During the first six-months of follow-up, the hospitalization rate was 10.2% for vaccinated individuals and 11.3% for unvaccinated individuals (p<0.001). After one-year of follow-up, the vaccinated group had a lower hospitalization rate (19.8% vs. 20.5%, p<0.001).

### Propensity score analysis

Due to the unequal distribution of baseline characteristics between the vaccinated and unvaccinated groups, propensity score analysis was performed. Figure 1 shows that most of the baseline characteristics were well balanced within each propensity quintile. During the first six months of follow-up, there was no significant difference in neurological and autoimmune diseases between the vaccinated and unvaccinated individuals using propensity analysis (Table 4). After one year of follow-up, the vaccinated individuals didn't incur higher risk for neurologic diseases or autoimmune diseases compared with the unvaccinated group after adjusting for other factors (Table 4).

After adjusting other factors, influenza vaccinations decreased the hospitalization rate to 19% for the first six-months of follow-up (OR, 0.81; 95% CI, 0.77–0.84) and 13% for one year of follow-up (OR, 0.87; 95% CI, 0.85–0.72).

## Discussion

Influenza epidemics occur yearly and have a significant impact on the health of the elderly and medical expenditures. Although public health vaccination is freely available in Taiwan, up to 55% of the elderly did not receive an influenza vaccination. Concerns about the adverse effects of influenza vaccination may be one of the barriers. More clinics visiting for URI symptoms, men, increased age, and number of comorbidies were independent predictors for those receiving an influenza vaccination. In this series, the increased risks were not evident for selected neurological and autoimmune diseases for the first-six months and one-year of follow up among the vaccinated individuals. Furthermore, influenza vaccination reduced the rate of all causes for hospitalization.

The strengths of our study are that it is based upon a large population (n = 93,049), the population experienced almost complete follow-up of neurological and autoimmune outcomes (99%) and had routine monitoring of diagnosis accuracy by the National Health Insurance Bureau of Taiwan. We found the elderly in Taiwan to have an influenza vaccination rate of 45%. There were also significant differences patient characteristics between the vaccinated and unvaccinated groups. Multiple logistic regression showed that more visiting clinics for URI symptoms in the prior 6 months, men, increased age, number of comorbidies, and residence in central/southern Taiwan were positive predictors for influenza vaccination. However, residence in urban/suburban areas and high SES were negative predictors for vaccination. These predictors, which are in agreement with previous series in Taiwan, could be used to find the target population in order to increase the future influenza vaccination coverage rate by the National Health Research Institution [18], [23]. The quality of the risk-adjustment technique when analyzing administrative information is an important issue. There is a significant difference in the baseline characteristics between the vaccinated and unvaccinated groups. Propensity scores were used to stratify the patients into five groups with similar scores in order to reduce the effects of selection bias between the vaccinated and unvaccinated groups [22], [24], [25]. Our results suggest that the influenza vaccine did not incur higher risk for neurological and autoimmune diseases. Influenza vaccination reduced the risk of hospitalization by 13% in elderly persons using propensity score analysis for one-year of follow-up. The effect of the vaccine on the hospitalization rate was more evident (19%) during the first six months than in the rest of the study period.

Several studies reported adverse events following influenza vaccination. After vaccination with the 1976 H1N1 influenza vaccine, 8.8 excess GBS cases per million vaccine doses were noted [9]. Substantial elevation of FBS risk within 6 weeks after vaccination and a low percentage of reporting other potential causes among the GBS cases supported the causal relationship between the influenza vaccination and GBS. A previous study reported that the H1N1 influenza vaccination incurred higher risk for Bell's palsy, which may be contributed to a phenomenon of Guillain-Barre syndrome [13]. However, other large-scale studies have questioned the association of Guillain-Barre syndrome and influenza vaccination [26], [27], [28]. Under the Emerging Infections Program (EIP) surveillance system for near-real time GBS surveillance throughout the 2009 to 2010 pH1N1 vaccination program, recent reports suggested that H1N1 influenza vaccine from 2009 to 2010 was associated with 0.8 excess GBS cases per million pH1N1 vaccines doses [29], [30]. The EIP system was featured by active case-finding and classification based on clinical criteria rather than administrative data codes alone, patient interviews for assessment of antecedent sources [31]. However, the lack of temporal clustering evidence and the possible exposure to other factors in over half of the GBS reports precluded the conclusion of the causal relationship between the H1N1 vaccine and GBS. In our series, there is a seasonal preponderance for GBS in both vaccinated and unvaccinated groups. 41% to 42% of GBS instances developed in the spring, which is in agreement with the report by Lyu et al [32]. After the six-months and one year of follow-up, our data did not reveal a statistically increased risk for Guillain-Barre syndrome in elderly individuals with an influenza vaccination.

Previous literature questioned the association of the influenza vaccination and autoimmune diseases [33], [34], [35]. Autoimmune diseases, such as reactive arthritis and polyarteritis nodosa, were reported after influenza vaccination. One possible mechanism is molecular mimicry when a structure similarity exists between viral antigens or other components of the vaccine. This phenomenon may trigger autoimmune responses [34]. Adjuvants were proven to reduce the amount of antigen, improve the magnitude, and prolong the duration of the immune response in H5N1 vaccines [10], [36]. However, lupus autoantibodies could be induced by adjuvants, such as the incomplete Freund's adjuvant, pristine, or squalene in vaccines [11], [12]. In our series, Fluvirin, Vaxigrip and Fluarix were all non-adjuvanted vaccines and there was no significant difference in autoimmune disorders between the vaccinated and unvaccinated individuals.

Up to 88% of elderly persons have one or more chronic conditions [37]. Chronic diseases or inflammation may be a risk factor for cardiovascular events, especially for elderly persons [38]. Systemic respiratory tract infections may aggravate this impending obstructive vessel and lead to acute myocardial infarction and stroke [39]. Poor physical function and impairment of judgment after influenza infection may result in falling or traffic accidents [40]. Poor compliance in the control of preexisting chronic diseases might exacerbate a disadvantaged condition, such as hypertension and diabetes. All of the above mentioned adverse effects could be erased by influenza vaccination. For the elderly, influenza vaccination could reduce hospitalization rates and shorten hospital stays [4], [5].

Barriers and predictors for influenza vaccination have been explored. Among the elderly, individuals with threat-responsiveness or perceived risk (prior influenza vaccination, or prior outpatient visits for flu-like respiratory conditions), men, persons aged 70 to 74 years, and those with chronic disease were more likely to have an influenza vaccination in Taiwan [18], [23]. Among the elderly, influenza vaccination coverage levels were about 65.6% to 66.3% between 2006 and 2009 in the United States, and this is still far away from the goal of Healthy People 2010 [14]. In Taiwan, the influenza vaccination coverage levels ranged from 38 to 50% [23], [41]. There is ample room for further vaccination rate increases among older individuals. Our series validated the protective effect of influenza vaccine, and the risks of adverse effects from the vaccine were not significantly elevated. Some strategies to support hospitalized patients, such as personalized postcards or phone calls, are effective. Home visits and facilitators may be effective in increasing influenza vaccination rates for the elderly in the community. Influenza vaccination programs for healthcare workers in hospitals or clinics is also worthy of a trail [42], [43].

Consideration must also be given to the potential limitations of the present study that may have influenced the observed conclusions. First, the diagnoses of neurological and autoimmune disease, and any other comorbid conditions, are completely dependent on ICD codes among the administrative data. However, the National Health Insurance Bureau of Taiwan randomly reviewed the charts and interviewed patients in order to verify the diagnosis. Hospitals with outlier chargers or practice could undergo an audit, with subsequent heavy penalties for malpractice or discrepancies. The diagnosis of GBS were further verified by the neurology specialists and the validity of coding for GBS in administrative data is confirmed [44]. The diagnosis of multiple sclerosis, rheumatoid arthritis and type I diabetes were confirmed by catastrophic disease dataset released from the NHIRD. Second, the database does not contain information on tobacco use, dietary habits, and body mass index, which may also be risk factors for neurological events and may contribute to hospitalization for cardiovascular diseases. We hope to further link the NHIRD with primary survey data in the future. Third, we used in-hospital mortality as a proxy of estimation of mortality rate. There may have been a few individuals with severe disease who chose not to seek medical help. However, the barriers to medical access is almost negligible because the National Health Insurance system in Taiwan allows patients to visit any clinic or hospital freely without referral by a general practitioner and cover up to 90% of the hospitalization costs.

In conclusion, 45% of the elderly aged 65 years or older received influenza vaccinations in 2008. Based on data from one year of follow-up, we did not find statistically increased risks for neurological and autoimmune diseases among the vaccinated individuals after adjusting for other factors. Influenza vaccinations decreased the risk for hospitalization by 13% in elderly patients during the one-year of follow-up. Based on these reports, we could try to provide more information based upon tangible evidence on influenza vaccination, particularly its effect on preventing epidemic outbreaks and the protective effects of hospitalization to those who do not think the vaccine is necessary and for those who are concerned about the adverse effects.

## References

[1] Fiore AE UT, Broder K, Finelli L, Euler GL, Singleton JA, et al.; Centers for Disease Control and Prevention (CDC) (2010) Prevention and control of influenza. Recommendations of the Advisory Committee on Immunization Practices (ACIP). MMWR Recomm Rep 59: 1–62. 20689501

[2] Thompson WW, Shay DK, Weintraub E, Brammer L, Bridges CB, et al (2003) Mortality associated with influenza and respiratory syncytial virus in the united states. JAMA: The Journal of the American Medical Association 289: 179–186. 12517228 10.1001/jama.289.2.179

[3] Thompson WW, Shay DK, Weintraub E, Brammer L, Bridges CB, et al (2004) Influenza-associated hospitalizations in the united states. JAMA: The Journal of the American Medical Association 292: 1333–1340. 15367555 10.1001/jama.292.11.1333

[4] Wang CS, Wang ST, Lai CT, Lin LJ, Lee CT, et al (2004) Reducing Major Cause-Specific Hospitalization Rates and Shortening Hospital Stays after Influenza Vaccination. Clinical Infectious Diseases 39: 1604–1610. 15578359 10.1086/425323

[5] Nichol KL, Nordin JD, Nelson DB, Mullooly JP, Hak E (2007) Effectiveness of Influenza Vaccine in the Community-Dwelling Elderly. New England Journal of Medicine 357: 1373–1381. 17914038 10.1056/NEJMoa070844

[6] Kao CD, Chen JT, Lin KP, Shan DE, Wu ZA, et al (2004) Guillain–Barré syndrome coexisting with pericarditis or nephrotic syndrome after influenza vaccination. Clinical Neurology and Neurosurgery 106: 136–138. 15003306 10.1016/j.clineuro.2003.11.002

[7] Chou CH, Liou WP, Hu KI, Loh CH, Chou CC, et al (2007) Bell's palsy associated with influenza vaccination: Two case reports. Vaccine 25: 2839–2841. 17084492 10.1016/j.vaccine.2006.10.006

[8] Mutsch M, Zhou W, Rhodes P, Bopp M, Chen RT, et al (2004) Use of the Inactivated Intranasal Influenza Vaccine and the Risk of Bell's Palsy in Switzerland. New England Journal of Medicine 350: 896–903. 14985487 10.1056/NEJMoa030595

[9] Schonberger LB, Bregman DJ, Sullivan-Bolyai JZ, Keenlyside RA, Ziegler DW, et al (1979) Guillain-Barre syndrome following vaccination in the National Influenza Immunization Program, United States, 1976–1977. Am J Epidemiol 110: 105–123. 463869 10.1093/oxfordjournals.aje.a112795

[10] Schwarz TF, Horacek T, Knuf M, Damman H-G, Roman F, et al (2009) Single dose vaccination with AS03-adjuvanted H5N1 vaccines in a randomized trial induces strong and broad immune responsiveness to booster vaccination in adults. Vaccine 27: 6284–6290. 19856521 10.1016/j.vaccine.2009.01.040

[11] Kuroda Y, Nacionales DC, Akaogi J, Reeves WH, Satoh M (2004) Autoimmunity induced by adjuvant hydrocarbon oil components of vaccine. Biomedicine & Pharmacotherapy 58: 325–337. 15194169 10.1016/j.biopha.2004.04.009

[12] Satoh M, Kuroda Y, Yoshida H, Behney KM, Mizutani A, et al (2003) Induction of lupus autoantibodies by adjuvants. Journal of Autoimmunity 21: 1–9. 12892730 10.1016/s0896-8411(03)00083-0

[13] Bardage C, Persson I, Örtqvist Å, Bergman U, Ludvigsson JF, et al.. (2011) Neurological and autoimmune disorders after vaccination against pandemic influenza A (H1N1) with a monovalent adjuvanted vaccine: population based cohort study in Stockholm, Sweden. BMJ 343. 10.1136/bmj.d5956 PMC3192001 21994316

[14] US Deparment of Health and Human Services (2000) Healthy people 2010. With understanding and improving health and objectives-full report, with commentary. Washington, DC.

[15] NHIRD (2009) Available: http//www.nhri.org.tw/nhird//date_01.html.Accessed 2009 Jan 2.

[16] Sheu JJ, Chiou HY, Kang JH, Chen YH, Lin HC (2010) Tuberculosis and the Risk of Ischemic Stroke. Stroke 41: 244–249. 20035070 10.1161/STROKEAHA.109.567735

[17] European Medicines Agency (2009) CHMP recommendations for the pharmacovigilance plan as part of the risk management plan to be submitted with the marketing authorisation application for a pandemic influenza vaccine. Report No EMEA/359381/2009 rev EMA, 2009.

[18] Tsai YW, Huang WF, Wen YW, Chen PF (2007) The relationship between influenza vaccination and outpatient visits for upper respiratory infection by the elderly in Taiwan. Value Health 10: 117–127. 17391420 10.1111/j.1524-4733.2006.00158.x

[19] Chou FHC, Tsai KY, Su CY, Lee CC (2011) The incidence and relative risk factors for developing cancer among patients with schizophrenia: A nine-year follow-up study. Schizophrenia research 129: 97–103. 21458957 10.1016/j.schres.2011.02.018

[20] Lee CC, Su YC, Ho HC, Hung SK, Lee MS, et al (2011) Risk of Stroke in Patients Hospitalized for Isolated Vertigo. Stroke 42: 48–52. 21127296 10.1161/STROKEAHA.110.597070

[21] Chang CM, Huang KY, Hsu TW, Su YC, Yang WZ, et al (2012) Multivariate Analyses to Assess the Effects of Surgeon and Hospital Volume on Cancer Survival Rates: A Nationwide Population-Based Study in Taiwan. PLoS ONE 7: e40590. 22815771 10.1371/journal.pone.0040590 PMC3398946

[22] Rubin DB (1997) Estimating causal effects from large data sets using propensity scores. Ann Intern Med 127: 757–763. 9382394 10.7326/0003-4819-127-8_part_2-199710151-00064

[23] Li YC, Liu CM (2009) Threat-responsiveness and the decision to obtain free influenza vaccinations among the older adults in Taiwan. BMC Public Health 9: 275. 19646246 10.1186/1471-2458-9-275 PMC2734847

[24] Rubin DB (1993) Tasks in statistical inference for studying variation in medicine. Med Care 31: YS103–110. 8492580 10.1097/00005650-199305001-00017

[25] D'Agostino RB Jr (1998) Propensity score methods for bias reduction in the comparison of a treatment to a non-randomized control group. Stat Med 17: 2265–2281. 9802183 10.1002/(sici)1097-0258(19981015)17:19<2265::aid-sim918>3.0.co;2-b

[26] Roscelli JD, Bass JW, Pang L (1991) Guillain-Barre syndrome and influenza vaccination in the US Army, 1980–1988. Am J Epidemiol 133: 952–955. 2028981 10.1093/oxfordjournals.aje.a115974

[27] Kaplan JE, Katona P, Hurwitz ES, Schonberger LB (1982) Guillain-Barre syndrome in the United States, 1979–1980 and 1980–1981. Lack of an association with influenza vaccination. JAMA 248: 698–700. 7097920

[28] Hurwitz ES, Schonberger LB, Nelson DB, Holman RC (1981) Guillain-Barre syndrome and the 1978–1979 influenza vaccine. N Engl J Med 304: 1557–1561. 7231501 10.1056/NEJM198106253042601

[29] Wise ME, Viray M, Sejvar JJ, Lewis P, Baughman AL, et al (2012) Guillain-Barré Syndrome During the 2009–2010 H1N1 Influenza Vaccination Campaign: Population-based Surveillance Among 45 Million Americans. American Journal of Epidemiology 175: 1110–1119. 22582209 10.1093/aje/kws196 PMC3888111

[30] Centers for Diseases Control and Preventotion (2010) Preliminary results: surveillance for Guillain-Barré syndrome after receipt of influenza A (H1N1) 2009 monovalent vaccine – United States, 2009–2010. MMWR Morb Mortal Wkly Rep 59: 657–661. 20520590

[31] Koobatian TJ BG, Schramm MM, Vogt RL (1991) The use of hospital discharge data for public health surveillance of Guillain-Barré syndrome. Ann Neurol 30: 618–621. 1789689 10.1002/ana.410300418

[32] Lyu RK, Tang LM, Cheng SY, Hsu WC, Chen ST (1997) Guillain-Barré syndrome in Taiwan: a clinical study of 167 patients. J Neurol Neurosurg Psychiatry 63: 494–500. 9343130 10.1136/jnnp.63.4.494 PMC2169759

[33] Aharon-Maor A SY (2000) The good, the bad and the ugly: vaccination. Isr Med Assoc J 2: 225–227. 10774273

[34] Shoenfeld Y A-MA (2000) Vaccination and autoimmunity-‘vaccinosis’: a dangerous liaison? J Autoimmun 14: 1–10. 10648110 10.1006/jaut.1999.0346

[35] Symmons DP, Chakravarty K (1993) Can immunisation trigger rheumatoid arthritis? Annals of the Rheumatic Diseases 52: 843–844. 8311532 10.1136/ard.52.12.843 PMC1005209

[36] Leroux-Roels I, Roman F, Forgus S, Maes C, De Boever F, et al (2010) Priming with AS03A-adjuvanted H5N1 influenza vaccine improves the kinetics, magnitude and durability of the immune response after a heterologous booster vaccination: An open non-randomised extension of a double-blind randomised primary study. Vaccine 28: 849–857. 19835828 10.1016/j.vaccine.2009.10.017

[37] Hoffman C, Rice D, Sung HY (1996) Persons With Chronic Conditions. JAMA: The Journal of the American Medical Association 276: 1473–1479. 8903258

[38] Elkind MSV, Luna JM, Moon YP, Boden-Albala B, Liu KM, et al (2010) Infectious Burden and Carotid Plaque Thickness. Stroke 41: e117–e122. 20075350 10.1161/STROKEAHA.109.571299 PMC2830875

[39] Smeeth L, Thomas SL, Hall AJ, Hubbard R, Farrington P, et al (2004) Risk of Myocardial Infarction and Stroke after Acute Infection or Vaccination. New England Journal of Medicine 351: 2611–2618. 15602021 10.1056/NEJMoa041747

[40] Barker WH, Borisute H, Cox C (1998) A Study of the Impact of Influenza on the Functional Status of Frail Older People. Arch Intern Med 158: 645–650. 9521230 10.1001/archinte.158.6.645

[41] Tsai YW, Huang WF, Wen YW, Chen PF (2007) The Relationship between Influenza Vaccination and Outpatient Visits for Upper Respiratory Infection by the Elderly in Taiwan. Value in Health 10: 117–127. 17391420 10.1111/j.1524-4733.2006.00158.x

[42] Thomas RE RM, Lorenzetti D (2010) Interventions to increase influenza vaccination rates of those 60 years and older in the community. Cochrane Database Syst Rev 2010 (9): CD005188. 10.1002/14651858.CD005188.pub2 20824843

[43] North Carolina Center for Public Health Preparedness (2010) The Public Health Wrokforce Assessement Report, North Carolina, 2004. Available: http://nccphp.sph.unc.edu/wfds_assess_rpts/Statewide.pdf. Accessed 2010 May 10.

[44] Kang JH, Sheu JJ, Lin HC (2010) Increased Risk of Guillain-Barré Syndrome following Recent Herpes Zoster: A Population-Based Study across Taiwan. Clinical Infectious Diseases 51: 525–530. 20642353 10.1086/655136

